# Responsible coordination of municipal health and care services for individuals with serious mental illness: a participatory qualitative study with service users and professionals

**DOI:** 10.1186/s12913-024-10999-w

**Published:** 2024-05-16

**Authors:** Jorunn Nærland Skjærpe, Tatiana Aleksandrovna Iakovleva, Marianne Storm

**Affiliations:** 1 Department of Public Health, University of Stavanger, Postbox 8600 FORUS, 4036 Stavanger, Norway; 2 University of Stavanger, Stavanger Business School, Postbox 8600 FORUS, 4036 Stavanger, Norway; 3 Faculty of Health Sciences and Social Care, Molde University College, Molde, Norway; 4 Research Department, Research Group of Nursing and Health Sciences, Stavanger University Hospital, Stavanger, Norway

**Keywords:** Service integration, Primary health care, Mental healthcare, Service providers, Qualitative design, Innovation, Severe mental disorder

## Abstract

**Background:**

Care coordination is crucial to ensure the health of individuals with serious mental illness. The aim of this study was to describe and analyze an inclusive innovation process for coordinating municipal health and care services for individuals with serious mental illness.

**Methods:**

We conducted café dialogues with professionals and service users with serious mental illness. The café dialogues engaged participants in conversation and knowledge exchange about care coordination, adressing topics of efficiency, challenges, and improvement. We used a responsible innovation framework to analyze the innovation process.

**Results:**

Responsible coordination requires promoting service users’ health and ensuring communication and mutual awareness between professionals. Individual-level factors supporting responsible coordination included service users knowing their assigned professionals, personalized healthcare services, and access to meaningful activities. Provider-level factors included effective coordination routines, communication, information exchange, and professional familiarity. Results reflect professionals’ and service users’ perspectives on efficient care coordination, existing challenges, and measures to improve care coordination.

**Conclusion:**

Café dialogues are an inclusive, participatory method that can produce insights into the responsible coordination of municipal health and care services for individuals with serious mental illness. The responsible innovation framework is helpful in identifying care coordination challenges and measures for responsible coordination.

## Background

Approximately one in eight individuals worldwide have a mental illness [1], with depressive disorders and anxiety disorders the most prevalent types [1, 2]. Many individuals with serious mental illness (SMI) require coordinated care to ensure and improve their health [1, 3, 4]. We use the term SMI to refer to schizophrenia, schizoaffective disorder, psychotic disorders, major depressive disorders, and bipolar disorders [5]. In Norway, approximately 1-3.5% of the population meets the criteria for lifetime SMI [2].

Care coordination is defined as “the deliberate organization of patient care activities between two or more participants (including the patient) involved in a patient’s care to facilitate the appropriate delivery of healthcare services” [6, p. 6]. Such coordination across services is essential to providing seamless healthcare [7]. Efficient care coordination relies on stakeholders sharing a common understanding and harnessing their skills, perspectives, experiences, and knowledge to address the needs of service users [8].

McDonald et al. [6] developed a framework of measures they deem essential for coordinated care, which has been applied in numerous studies on mental healthcare coordination [3, 9, 10]. The measures can be grouped into individual, provider, and system levels of care [6, 9, 11, 12]. At the individual level, professionals tailor service delivery to meet service users’ needs [6]. Care coordination involves assessing individuals’ healthcare needs, resources, and goals, providing personalized services, supporting self-care, and encouraging health-promoting activities in the community [6]. At the provider level, care coordination involves establishing accountability, negotiating responsibility for service users’ care, allocating tasks and responsibilities, organizing patient transitions between services, and exchanging information among professionals and service users [6]. Coordination routines, communication platforms, and cooperative relationships are also important at this level [8, 13, 14]. Care coordination at the system level focuses on aligning healthcare and community resources with the population’s needs [6], health policy goals, economic factors, legislation, and regulations influencing professional action and decision-making [14].

Several challenges affect mental healthcare coordination [4]. These include a lack of access to services, issues with information exchange, and limited service user involvement in decision-making [4]. The latter is due to an asymmetrical power balance between professionals and service users and perceptions that individuals with SMI lack the capacity to be involved in decision-making due to their symptoms [15]. Moreover, professionals can at times act unilaterally in service users’ best interests or struggle to integrate their expertise with service user insights, making service users feel unheard or defined by diagnostic labels [15]. Efficient care coordination can also be hindered by professionals’ multiple simultaneous tasks, uncertainty regarding task prioritization, prioritizing treatment over prevention, and a deficit of expertise [16]. Knowledge differences, conflicts, and difficulties with perspective-taking are additional challenges [17]. Addressing these issues requires a more symmetrical power balance and ensuring equal influence of professionals from different services [17, 18] and service users [15, 17].

Improved care coordination is a stated health policy goal in Norway and internationally [19–21]. One way to achieve this goal is through responsible innovation processes that include stakeholders [22]. We define responsible innovation as “taking care of the future through collective stewardship of science and innovation in the present” [23, p. 1570]. Stilgoe et al. [23] present a framework for responsible innovation with four integrated dimensions: inclusion, anticipation, reflexivity, and responsiveness. *Inclusion* refers to encouraging different perspectives, anchoring decisions, and promoting reflective innovation processes. Inclusion can occur through dialogues and by applying stakeholders’ ideas and knowledge. *Anticipation* is about assessing what is known, what is likely to occur, what one intends to achieve, how to address relevant issues, and discovering additional opportunities for innovation. *Reflexivity* integrates stakeholder perspectives about expectations, challenges, and other issues. *Responsiveness* refers to an open and flexible innovation process in which participants respect and adapt to each other’s perspectives and knowledge. Suitable responses may involve measures that address existing issues and potential future challenges.

This study builds upon literature demonstrating that responsible innovation and the inclusion of diverse stakeholders can effectively improve healthcare services [22, 24–27]. We include professionals and service users with SMI in café dialogues, a participatory research method [28–33], to identify care coordination challenges, address shared coordination responsibilities, and develop improvement measures [34–36]. In health and social science, such participatory research methods have successfully captured varied perspectives and found sustainable solutions to challenges [31, 37–42]. Service users have valuable knowledge based on their experiences, can offer insights into their needs and challenges, and can suggest potential improvement measures [24, 43]. Involving professionals in café dialogues can enhance their understanding of service users’ perspectives and improve interactions between service users and healthcare services [44]. The aim of this study is to describe and analyze an inclusive innovation process for coordinating municipal health and care services for individuals with SMI.

## Methods

### Study setting: the Norwegian healthcare system

The Norwegian healthcare system consists of municipal health and care services and specialist health services. Municipalities are responsible for delivering primary healthcare services outside of hospital facilities. The municipal health and care services focus on disease prevention, health promotion, treatment, care, and assistance with daily life functions [45]. Municipal health and care services for people with SMI encompass general practitioners (GPs), emergency rooms, inpatient acute care, institutions, home-based healthcare, and supported housing with round-the-clock healthcare [45]. GPs serve as gatekeepers and coordinate referrals to municipal health and care services and specialist health services as per service users’ needs [45].

The study was conducted in municipal health and care services for individuals with SMI in a rural Norwegian municipality with nearly 20,000 inhabitants. Individuals with SMI frequently experience persistent and severe symptoms of mental illness [4, 46, 47]. They often have extensive physical healthcare needs and a higher mortality rate than the general population [2, 48–51]. Their life expectancy is up to 30 years shorter, mainly because of physical health issues [48–50, 52].

The severity of each individual’s mental health issues is based on their symptoms, challenges, and impact on social and daily functioning [53]. Severity determines the primary responsibility for care, services provided, level of care coordination required, and involvement of specialist health services [53]. If municipalities lack sufficient competence to meet service users’ needs, responsibility falls to specialist health services which, for individuals with SMI, encompass inpatient and outpatient treatment and care offered by psychiatric hospitals and community mental health centers [54]. Individuals with SMI often receive municipal and specialist health services [53].

### Study design, recruitment, and participants

For this study, we employed a qualitative participatory design [55] to describe and analyze an inclusive innovation process for coordinating municipal health and care services for individuals with SMI. Café dialogues were used to collect data [28–33]. Café dialogues are well suited to explore topics and obtain knowledge through varied participant perspectives in a relatively short period [28–33]. Café dialogues were considered well suited for several reasons, including their potential for obtaining qualitative data, broadening the reference sample size, exploring topics, and enhancing the diversity of perspectives on the given topic [31–33]. Additionally, they provide an arena for participants to share their reflections on lived experiences and develop new ideas [28–33]. This inclusive approach ensures that the participants’ voices and perspectives are included in the innovation and research process [23, 28–30].

We used purposive sampling [55] to recruit professionals and service users who were knowledgeable about care coordination and had experience with mental healthcare services. Recruiting a diverse range of professionals and service users aligns with the inclusion strategy of responsible innovation [23]. Our inclusion criteria for professionals were that they were employed in a municipal health and care service and were involved in care coordination and service delivery for individuals with SMI. For service users, inclusion criteria were that individuals currently or previously had a diagnosis of SMI and had received two or more municipal health and care services.

We recruited professionals through municipal health and care service leaders who were contacted by JNS via e-mail with information about the study. Service leaders then provided employees with this information and selected employees to participate based on willingness and interest. Service users were recruited through municipal health and care employees who were given information about the study by JNS via e-mail. These employees provided eligible participants with information about the study and the time and location of the café dialogue. Thirty professionals and seven service users were recruited for three café dialogues. Table 1 presents participants’ characteristics.

**Table 1:** Participant characteristics

Participant characteristics		Café dialogue 1 Professionals (*N* = 16)	Café dialogue 2 Professionals (*N* = 14)	Café dialogue 3 Service users (*N* = 7)
Gender	Female	15	13	6
	Male	1	1	1
Age (in years)	22–39	7	4	1
	40–49	5	6	2
	50–69	4	4	4
Employed in	Supported housing with round-the-clock healthcare	2	4	
	Municipal inpatient acute care	0	3	
	Mental healthcare institution	2	4	
	Mental health home care	10	0	
	Home nursing	1	2	
	Emergency room	1	1	

### Café dialogues

JNS and MS conducted three café dialogues, two for professionals and one for service users, in May and June 2021. Café dialogues were inspired by the dialogue seminar method described by Storm [28–30] and the World Café method [31–33], which are participatory methods for engaging participants in brainstorming, conversation, and knowledge exchange [28–33]. Both methods, emphasizing diverse perspectives and mutual learning among participants, offer insights for scientific inquiries and changes and promote inclusivity [28–33]. These methods facilitate knowledge exchanges and constructive dialogue by encouraging exploration and discussion of important questions [28–33]. They are adaptable to various contexts and purposes, with event invitations, designs, and questions tailored to specific circumstances [28–33]. Brown & Isaacs [32] outline seven design principles for the World Café, including setting the context, creating a welcoming environment, exploring meaningful questions, encouraging contributions from all participants, connecting diverse perspectives, listening collectively for insights, and sharing discoveries as a group.

The café dialogues took place in meeting rooms provided by the municipality. These rooms represented a neutral space where participants could feel free to engage with each other [32]. Each café dialogue lasted for two hours with a break halfway. We started each café dialogue by welcoming participants, introducing ourselves, and presenting the agenda for the day. We gave a 30-minute teaching session on care coordination to establish a shared understanding of the concept [25, 26], using PowerPoint slides to help explain care coordination [6], relevant health policy [19], and literature [56, 57] that highlighted the importance of care coordination and the complexities of ensuring and improving the health of individuals with mental illness. Following this, we facilitated brainstorming and knowledge exchange conversations where participants articulated their perspectives on the topics of efficient care coordination, coordination challenges, and measures to improve care coordination.

### Data collection

JNS led the café dialogues and took notes on a whiteboard throughout the sessions. MS took more detailed written notes to document participants’ ideas and knowledge. When necessary, we adjusted the dialogue to elicit participants’ perspectives on our topics of interest [23]. An important aspect was ensuring active participation and including each person’s perspectives in the innovation process [23]. All notes were subsequently synthesized into 13 pages of written text, constituting the qualitative empirical data material [55]. Table 2 illustrates the topics and activities of the café dialogues.

**Table 2:** Café dialogue topics and activities

Topics		Activities
Efficient care coordination Coordination challenges Measures to improve care coordination	Responsible coordination	Introduction Teaching session Brainstorming Knowledge exchange Lunch and coffee break

### Data analysis

We conducted inductive and deductive thematic analyses [58, 59]. For the inductive aspect, we analyzed the written notes from the café dialogues, focusing on participants’ perspectives on efficient care coordination, challenges, and improvement measures. The inductive analysis consisted of six stages: (1) reading through the material several times to become acquainted with it (2), coding meaning units relevant to the study aim (3), identifying themes and assigning meaning units to these themes (4), conducting a critical review of each theme to ensure that meaning units and themes comprehensively represented the data (5), labeling themes, and (6) summarizing into two themes and five sub-themes. Table 3 gives examples of the inductive thematic analysis.

For the deductive aspect, we analyzed the café dialogue innovation process by connecting themes and sub-themes from the inductive analysis to the dimensions of inclusion, anticipation, reflexivity, and responsiveness in Stilgoe et al.‘s [23] framework for responsible innovation.

#### Author reflexivity

Our understanding is built on the fact that we already have a relationship with the phenomena of coordination of healthcare services, and we can better understand existing concepts by relating reflexively to them [60]. In this study, reflexivity was embraced through collaborative efforts among the three authors in the data analysis [55, 61]. Each author, with distinct educational backgrounds and professional experience, brought different perspectives to the analysis, stimulating varied interpretations during the theme development process. We considered alternative interpretations before reaching a consensus on results that aligned with the study’s aim. Consequently, themes were not influenced solely by the preunderstanding of a single researcher, thereby enhancing the trustworthiness of the results [62].

**Table 3:** Examples of inductive thematic analysis

Meaning units	Codes	Sub-themes	Theme
It can feel unsettling to have professionals come to your home when you are experiencing substantial symptoms of anxiety and depression.	Home care distress	To be met and followed up with personalized healthcare	Ensuring and promoting health - Responsible coordination at the individual level
We must take control of our own lives. It can take a long time to realize that something needs to change. Waiting for someone else to fix me does not work. That keeps us stuck in the same old mess.	Personal responsibility		
Some service users may have needs beyond standardized services. In such cases, we must be curious, stretch ourselves, think outside the box, prioritize the most critical aspects, try new measures, and find solutions together.	Beyond standardized healthcare services	A balance between providing healthcare and allowing service users to take responsibility for their own lives	
It is a delicate balance to know how much help to provide while not assuming responsibilities that service users can handle themselves.	Assistance versus responsibility		

### Ethical considerations

The study was approved through Sikt– the Norwegian Agency for Shared Services in Education and Research (formerly known as the Norwegian Centre for Research Data, or NSD) (project No. 132714). Sikt ensured that the research project adhered to ethical guidelines and regulations and approved information security and privacy services as part of the Norwegian Directorate for Higher Education and Skills (HK-dir). The study adhered to the principles of the Helsinki Declaration. All participants took part voluntarily and were provided with information regarding confidentiality. They were informed that participation was voluntary and that they retained the right to withdraw from the study without consequences. Written informed consent was obtained from all participants before the café dialogues.

## Results

The thematic analysis resulted in two themes and five sub-themes that reflected responsible coordination at individual and provider levels, participants’ anticipation of efficient care coordination, reflexivity to care coordination challenges, and responsiveness to improvement measures [23].

At the individual level, anticipation reflected a desire that services be personalized, with relationships built on trust and security between professionals and service users. Reflexivity encompassed care coordination challenges, which arose when service users refused healthcare or did not recognize their healthcare needs. Suggested improvement measures included activity centers, flexible healthcare, and professionals taking time to get to know service users.

At the provider level, anticipation involved clear responsibilities and tasks, coordination routines, and communication. Professionals described care coordination challenges related to a lack of routines, communication, unfamiliarity with one another, absence of shared health record-keeping systems, disagreements regarding service users’ healthcare, and not completing expected tasks. Improvement measures were responses related to information exchange, meeting points for communication, and a shared health record-keeping system across services.

Themes and sub-themes are described in more detail below. Table 4 illustrates themes, sub-themes, and the café dialogue innovation process.

**Table 4:** Themes, sub-themes, and the innovation process

Inclusion in responsible coordination		Anticipation	Reflexivity	Responsiveness
Theme	Sub-theme	Efficient care coordination	Care coordination challenges	Improvement measures
1: Ensuring and promoting health - Responsible coordination at the individual level	1: To be met and followed up with personalized healthcare	Service users - are met in a way they feel respected, seen, and heard - are offered flexible and personalized services - take responsibility for their health, improvement, and life	Challenging for service users to receive healthcare	Professionals - provide healthcare in safe environments - adapt healthcare to the needs of service users (for example, at home, out, or in the office, or via text messages and video calls) - make it easy for service users to contact them (for example, through direct telephone numbers to GP or other familiar professionals) - use humor Double appointments with GP
	2: A balance between providing healthcare and allowing service users to take responsibility for their own lives	Professionals - adapt services to service users’ goals, resources, preferences, and healthcare needs - assist service users in managing their health	Challenging for professionals to meet service users’ needs Service users - are unable to always ensure their health - do not always receive healthcare - only accept healthcare from specific professionals - have a different perspective on their needs than professionals’	Professionals - from different services, find solutions to challenges together - try new measures as needed - prioritize the most critical aspects of care
	3: To know professionals and have access to meaningful activities	Service users - know the professionals involved - have access to meaningful activities Relationships built on trust and security among professionals and service users	Lack of access to meaningful activities Service users are not familiar with the professionals involved	Professionals - spend time getting to know service users - traveling on tour with service users (e.g., wekeend getaways) Service users - challenges themselves to participate in activities - partake in activity centers
2: Communication and knowledge of each other - Responsible coordination at the provider level	4: Coordination routines and information exchange	Professionals have - clearly defined responsibility areas and tasks - regular communication and information exchange Service users - are provided with personalized information - are involved in adapting their service offerings	Lack of coordination routines and exchange of information Service users - may require multiple services to ensure and improve health - do not consent to information exchange among professionals Professionals - find it challenging to determine the most suitable services for proper care - are under time pressure - disagree about service users’ healthcare - can not fulfill their expected tasks Lack of shared health record-keeping system across services	Professionals - have access to service users’ health records - develop care plans outlining service users’ allocated services and existing measures Service users - are provided with detailed written and oral information - have access to quality-assured information about health, illness, and healthcare options Simplify the process of service users giving consent to information exchange. Access to shared health record-keeping system across services
	5: Being familiar with cooperating professionals	Service users experience efficient care coordination Regular care coordination meetings	Involved professionals do not always know each other	Common meeting points for service users and involved professionals Vary meeting locations so professionals become acquainted with each other’s workplaces. Digital and hybrid meetings

### Theme 1: Ensuring and promoting health - Responsible coordination at the individual level

#### Sub-theme 1: To be met and followed up with personalized healthcare

Service users agreed that efficient care coordination required personalized healthcare with user involvement at every stage of their health journey. It can be challenging for service users to receive healthcare, even when they know their needs. When professionals approach service users in a way that makes them feel respected, seen, and heard, cooperation is easier. A service user elaborated on this:

*It is crucial that professionals do not adopt a top-down attitude and that they communicate clearly without using complicated terminology. We feel stupid and hesitant to speak up when we do not understand what the professionals are saying.*

Service users appreciated when healthcare was flexible and provided in environments where they felt safe. Service users suggested measures such as follow-ups at professionals’ offices, at home, or outdoors during activities that offered new experiences. They also mentioned video calls or double appointments with GPs. Using humor, even in serious situations, was seen as beneficial.

Several service users found communicating with professionals in writing easier than by phone. Both service users and professionals shared positive experiences using text messages and wanted to continue such measures. The ability to quickly and easily get in touch with professionals when needed was important for service users, and made easier through having direct numbers for their GP and other familiar professionals. Still, while these personalized and flexible measures were practical, service users emphasized that they were responsible for their health and that improved health required major effort on their part. One of them stated:

*We must take control of our own lives. It can take a long time to realize that something needs to change. Waiting for someone else to fix me does not work. That just keeps us stuck in the same old mess. Having services available does not help if we are not willing to change.*

#### Sub-theme 2: A balance between providing healthcare and allowing service users to take responsibility for their own lives

Professionals believed efficient care coordination involved personalized services adapted to service users’ goals, resources, preferences, and needs. One professional stated:

*Some service users may have needs beyond standardized services. In such cases, we must be curious, stretch ourselves, think outside the box, prioritize the most critical aspects, try new measures, and find solutions together.*

Several professionals noted that some service users needed assistance in managing their health. In such situations, efficient care coordination meant liaising with necessary services and assisting service users with daily needs. Professionals acknowledged that service users were responsible for their own lives but did not want to place too much pressure on them. One professional said:

*It is a delicate balance to know how much help to provide while not assuming responsibilities that service users can handle themselves.*

Professionals faced challenges in meeting service users’ needs if they refused healthcare, only accepted it from specific professionals, or had a different perspective on their needs compared to professional recommendations. These needs were often related to mental health but also to physical health. In such situations, it was essential for professionals to explain what was being refused.

#### Sub-theme 3: To know professionals and have access to meaningful activities

For service users, familiarity with professionals was crucial for efficient care coordination. Relationships built on trust and security, and professionals who took the time to get to know them made it easier to receive healthcare. Measures such as weekend getaways, holiday trips, and other meaningful activities were some suggested ways to get acquainted and build relationships. Engagement in meaningful activities promoted health, shifted focus away from illness, and placed a greater emphasis on service users’ strengths and resources. Conversely, a lack of meaningful activities posed coordination challenges.

Service users praised an activity center, often recommended by professionals, that helped establish contact. The activity center provided daily structure, social interaction, friendships, and belonging, helping service users discover they were not alone in their struggles and could support each other. In addition, they could receive support from peers and professionals, despite some instances of gossip and rumors among center users. Still, they were conscious of promoting each other positively and apologized if they said something they regretted. The center organized weekly group outings, which service users described as health-promoting and involving physical activity and fresh air, which, in turn, led to improved sleep and reduced symptoms of depression. Overall, attending the activity center had resulted in several service users needing less healthcare. One service user said:

*I went to my GP every three months before– now I only go every six months.*

While service users knew that such activities benefited them, actually engaging in such endeavors could still prove difficult. Nevertheless, doing so could instill a sense of accomplishment. One person spoke about challenging oneself:

*It is important to stand up for oneself, take responsibility, and dare to set limits. To have the courage to say both yes and no. If you say yes to something that feels daunting, it is crucial to be able to step back when needed. Trying things that can be enjoyable and beneficial for oneself is essential.*

### Theme 2: Communication and knowledge of each other - Responsible coordination at the provider level

#### Sub-theme 4: Coordination routines and information exchange

Professionals reported that individuals with SMI often have complex needs and require multiple services. Determining priorities for mental or physical healthcare needs and identifying suitable services could be challenging. An absence of coordination routines, especially between mental and physical healthcare services, and coordination efforts being affected by personal and time constraints made it difficult for professionals to address complex needs. There were also challenges when professionals disagreed about service users’ healthcare and when they could not fulfill their expected tasks. Therefore, clarifying care responsibilities for each involved service was crucial.

Communication and regular information exchange were seen as elements of efficient care coordination. Information exchange often occurs through an electronic messaging system. However, several professionals have experienced issues with this system, such as messages not being sent as intended. Also, written information sometimes can fail to convey nuances present in face-to-face communication, leading to misinterpretation. In such cases, professionals needed to consult colleagues or messengers to ensure they had correctly understood the information. One professional said:

*The information we receive and when it arrives can be quite random. Messages often get lost, or service users must provide the information themselves. Therefore, we spend extra time making calls and searching for necessary information.*

Service users wanted access to updated health information and professionals to exchange relevant information. This exchange was contingent upon service users’ consent. They also wanted to simplify the consent process. Professionals discussed service users who do not consent to information exchange or withdraw their consent, citing this as a challenge hampering healthcare. They were committed to explaining to service users the importance of information exchange and the consequences of withholding consent.

For professionals, efficient care coordination meant providing service users with regularly updated information so they could be involved in adapting services to their needs. Service users valued professionals providing comprehensive information about available services and the consequences of choosing or not choosing them, as they could easily forget this information. Therefore, detailed written and oral information and access to quality-assured information about health, illness, and healthcare options could be helpful. Service users often had to deal with unfamiliar professionals who lacked updated health information about them. One explained:

*Interacting with many professionals can be challenging when you feel emotionally unstable. It is exhausting to keep track of things and repeatedly explain your history when experiencing severe symptoms, such as telling professionals in the municipality what hospital doctors have said.*

Consequently, service users wanted involved professionals to have access to their health records. However, care coordination challenges arose from an absence of up-to-date health information, primarily attributed to the lack of shared health record-keeping across services. Professionals reported positive experiences creating plans that outlined allocated services and described existing measures for when service users experienced severe symptoms. These plans could be used independently of a shared health record system.

#### Sub-theme 5: Being familiar with cooperating professionals

Professionals stated it was important that service users experienced efficient care coordination. They noted that care coordination worked best when involved professionals were familiar with each other, with challenges more likely to arise if they were not. Service users wished for involved professionals to cooperate closely and be familiar with each other’s roles. One service user said:

*Professionals must clearly define their responsibilities so it is evident who does what. For example, we need to know who is responsible for our medications and who accompanies us to social services.*

Professionals highlighted regular care coordination meetings in the municipality and with specialist health services as crucial measures for efficient care coordination. These meetings could be used for professionals to get to know each other, allocate responsibilities and tasks, develop and revise coordination routines, ensure information exchange, and verify information was understood. Meetings between professionals from mental health home care and home nursing were particularly relevant for coordinating mental and physical healthcare. Meeting locations could vary so that professionals would become acquainted with each other’s workplaces. One professional said:

*Having common meeting points - meeting in person and talking together - makes it easier to initiate contact and know whom to reach out to electronically or by phone on future occasions.*

The municipality has tried digital and hybrid meetings. Several professionals found these formats useful and reported receiving positive feedback from service users. Such meetings save time as participants can attend only the parts relevant to them. Digital meetings also eliminate the need for travel.

## Discussion

In this study, we conducted café dialogues with professionals and service users with the aim of outlining an inclusive innovation process for the responsible coordination of municipal health and care services for individuals with SMI. Our study highlights how including professionals and service users can help improve responsible care coordination through clarifying anticipation, encouraging reflexivity, and ensuring responsiveness [23]. Furthermore, we discuss our results in light of the dimensions in Stilgoe et al.‘s [23] framework for responsible innovation and previous research.

According to Stilgoe et al.‘s [23] framework, clarifying participant anticipation is central to responsible innovation. Our findings show that at the individual level, anticipation for efficient care coordination includes promoting health through personalized and flexible healthcare services and service users taking responsibility for their own lives. Balancing these anticipations can, however, prove challenging for professionals. Service user involvement in care coordination can promote health and reduce illness symptoms [63], but research indicates that some individuals with SMI prefer professionals to make care coordination decisions on their behalf [10]. Continuous assessment of service users’ capacities is therefore crucial to ensure any responsibilities they are given are appropriate [64].

Reflexivity to challenges, their causes, and how they might impact participant anticipation are essential in Stilgoe et al.‘s framework [23]. In our study, consideration of reflexivity revealed that professionals at times faced coordination challenges in meeting service users’ health needs when users did not accept or recognize these needs. Research shows that such challenges are common in care coordination [3, 10, 65].

At the provider level of responsible coordination, Stilgoe et al.‘s [23] anticipation dimension involves efficient communication and information exchange among professionals. These are essential for successful care coordination [66] and meeting service users’ needs [14, 63]. Our study revealed that electronic messages were a preferred form of communication. Electronic communication can enhance efficient care coordination by providing quick access to up-to-date information [67]. However, participants reported communication challenges at the provider level, as problems with electronic messages resulted in professionals missing vital information and having to spend time gathering it. Further, electronic messages may not always suffice as essential information and details can be omitted or misunderstood [10, 67].

Considering reflexivity at the provider level, we identified challenges whereby some service users declined to consent to information exchange among professionals, likely due to privacy concerns [67]. A lack of consent can limit shared health record-keeping, proving a challenge for coordination and weakening the quality of electronic information [66]. We found that service users found it burdensome to repeat their history when interacting with different professionals, an observation supported by prior research [68]. Shared health record-keeping can be particularly valuable if service users struggle to express themselves [67].

In our study, Stilgoe et al.‘s [23] responsiveness dimension represented measures to improve care coordination. Participants described measures for responsible coordination that focused on flexible and personalized services, such as simplifying contact with professionals, providing healthcare in a safe environment, and professionals getting to know service users. Customized services are vital in care coordination [14] and essential to meeting service users’ mental and physical health needs [10].

Our results suggest that improvement measures at the individual level should allow service users to communicate their needs to professionals in writing. Text messages could be used in personalized healthcare to quickly convey health information, increasing service user involvement and supporting self-care and recovery [69]. Our results highlight the use of activity centers as an improvement measure as these promote service users’ health and reduce the need for healthcare. Access to health-promoting services can help to engage individuals through shared interests and experiences, which is particularly important when service users have complex needs, as other needs may take precedence over health promotion [70].

Professionals in our study stated that lack of care coordination and unclear responsibilities made it challenging to determine the most suitable services for service users with complex needs. They responded to these challenges by developing coordination routines. This aligns with the literature indicating that efficient care coordination at the provider level depends on measures such as routines and transparent allocation of responsibilities and tasks [14, 66]. Our results show that care plans that outline allocated services and measures accessible to professionals are important for efficient care coordination, supporting previous findings [66].

Service users in our study appreciated when involved professionals knew each other and highlighted the importance of having familiar professionals present. Considering the issue of responsiveness, participants reported that regular meetings allowed professionals to get to know each other, establish routines, communicate, and distribute tasks and responsibilities. Meetings where participants work toward common goals are essential for efficient coordination [3, 9, 10, 63] and complement the care coordination process [8]. Our results show that digital and hybrid meetings increase meeting participation, with video meetings saving time and improving accessibility [71]. Research indicates that service user participation in care coordination meetings ensures that services align with the users’ wishes, needs, resources, and goals [63].

### Strengths and limitations

A key strength of this study was the inclusion of service users and professionals from multiple municipal health and care services. The café dialogue method encouraged the integration of these participants’ perspectives into the innovation process [23, 44]. During the café dialogues, participants discussed various aspects of care coordination. They were able to relate the teaching sessions to their own experiences. Additionally, they highlighted their own examples of coordinated care. Participants were willing to share their personal experiences, and the dialogues enabled them to build upon each other’s responses [31, 55, 72], fostering inclusiveness [23] and relevance to practice. Several participants offered valuable ideas and knowledge about how to improve care coordination [23, 24].

However, some participants showed less engagement in conversations, and some required direct questions before sharing their perspectives. This may have been due to varying attitudes toward innovation, with some participants more passive and conservative and others more open to new ideas [25]. The teaching session at the café dialogues could have influenced participants’ conversations. It is also possible that relevant voices were not represented in our recruitment approach. The café dialogue for service users was conducted in a meeting room with access to professionals the users knew well and could contact if needed. We note that as participants were encouraged not to share experiences of a sensitive and private nature, some service user participants may have been reluctant to share opinions or negative experiences due to privacy concerns.

Looking back, we realize that audio recording of the café dialogues could have captured the participants’ viewpoints more thoroughly and preserved their ideas and knowledge. Furthermore, employing an interview guide covering a wider range of topics than those discussed in the dialogues could have enhanced our understanding of care coordination.

Due to constraints imposed in response to the COVID-19 pandemic, most participants in the first café dialogue were employed in mental health home care. Another potential limitation is that service users were a minority of our participants. Due to pandemic-related restrictions, we could not include more service users, and professionals and service users were not allowed to participate in the same café dialogue. In future research, we recommend including more service users to balance the number of service users and professionals. We also suggest that both professionals and service users participate in the same café dialogues. This could generate varied dynamics and potentially result in alternative suggestions for improvements. Finally, participants’ suggestions were not put into practice, so their impact on the improvement and coordination of services remains unknown.

## Conclusion

This study shows that café dialogues that include professionals and service users are an inclusive, participatory method that can inform the responsible coordination of municipal health and care services for individuals with SMI. Our results indicate that responsible coordination at the individual level entails ensuring and promoting service users’ health, while at the provider level, communication and knowledge of each other are central. Findings demonstrate the relevance of the responsible innovation framework in identifying care coordination challenges and its utility in developing measures for responsible coordination of municipal health and care services for those with SMI. These results may be transferable to other contexts.

## Data Availability

Data are available to appropriate academic parties upon reasonable request to the corresponding author.

## Abbreviations

GP: General practitioner
HK-dir: Norwegian Directorate for Higher Education and Skills
NSD: Data Protection Official for Research at the Norwegian Centre for Research Data
SMI: Serious mental illness
